# Distal anterior interosseous nerve to ulnar nerve motor branch reverse end-to-side transfer in a case of severe ulnar neuropathy

**DOI:** 10.3171/2022.9.FOCVID2282

**Published:** 2023-01-01

**Authors:** Rajesh Krishna Pathiyil, Kate Elzinga, Daniel Umansky, Rajiv Midha

**Affiliations:** 1 Departments of Clinical Neurosciences and; 2 Plastic Surgery, University of Calgary, Alberta, Canada

**Keywords:** reverse end-to-side transfer, severe ulnar neuropathy, anterior interosseous nerve to ulnar nerve transfer

## Abstract

Reverse end-to-side (RETS) distal transfer is gaining popularity in cases of proximal nerve damage with the nerve in continuity, allowing the nerve to potentially retain its ability to regenerate and recover. While preserving the original axon pool, RETS could provide an additional pool of motor axons and/or possibly "babysit" the muscle endplates and distal denervated nerve Schwann cells until reinnervation from the original pool occurs. The authors present a video demonstrating anterior subcutaneous transposition of the ulnar nerve at the elbow coupled with a distal anterior interosseous nerve to ulnar nerve RETS in a case of severe posttraumatic ulnar neuropathy at the elbow.

The video can be found here: https://stream.cadmore.media/r10.3171/2022.9.FOCVID2282

## Transcript

This is Dr. Rajiv Midha and I am pleased to present a case of a distal anterior interosseous nerve (AIN) transfer^1–4^ in this patient with severe traumatic ulnar neuropathy at the elbow.

### 0:31 Clinical Presentation of the Patient.

This patient’s physical exam, as you will see in a moment, showed complete loss of hand intrinsic motor function of the ulnar nerve along with dense sensory involvement and only a flicker of movement at the flexor digitorum (profundus) to the little finger.

### 1:00 Clinical Examination.

[The patient is being asked to flex the wrist, and to flex, extend, abduct, and adduct the digits.]

### 1:26 Plan: Left Ulnar Nerve Decompression at the Elbow and Distal Anterior Interosseous Nerve to Ulnar Nerve Motor Branch Reverse End-to-Side Transfer (RETS).

In most patients who have ulnar nerve pathology at the elbow, such as compression with motor dysfunction, we would normally do a simple in situ decompression. In this patient who had significant trauma both in the soft tissue and the joint and bones, we chose to do an ulnar nerve transposition in the subcutaneous position for the reasons identified in the slide. As you will see in the subsequent videos, we did a very thorough decompression and neurolysis of the ulnar nerve from well above the elbow joint to well below the elbow joint through the two heads of the flexor carpi ulnaris.

### 2:12 Positioning.

The patient was positioned supine under general anesthesia, with the arm on a plastics arm board, and prepared and draped from distal axilla down to hand.

### 2:23 Incision for Ulnar Nerve Decompression and Anterior Subcutaneous Transposition at the Elbow.

The incision at the elbow was curvilinear, centered at the medial epicondyle and just posterior to it.

### 2:30 Decompression of Ulnar Nerve in the Cubital Tunnel.

There was significant scarring and adhesions around the ulnar nerve, which were dissected using sharp techniques, with the ulnar nerve mobilized with external neurolysis, first above the elbow and then sequentially, at the elbow joint and then distally. We identified and preserved a large branch from the medial antebrachial cutaneous (MABC) nerve. Then we used further sharp dissection to expose and unroof the distal ulnar nerve as it went into the two heads of the flexor carpi ulnaris (FCU) muscle and deep to them. We identified both an articular branch, which we subsequently sacrificed, and an important branch to the FDP (flexor digitorum profundus) and FCU more distal, which was preserved in continuity along with the main trunk of the ulnar nerve. This allowed a generous length of the ulnar nerve. We stimulated the ulnar nerve at the elbow and found that there was no contraction of the muscles of the intrinsic hand, but there was some contraction of the FDP to the little finger.

### 3:47 Anterior Subcutaneous Transposition of the Ulnar Nerve at the Elbow.

We then resected the medial intermuscular septum so that we could transpose the ulnar nerve in the subcutaneous plane. To do this, we lifted off a large amount of subcutaneous tissue of the flexor pronator mass, placed the ulnar nerve deep to the subcutaneous tissue, and sutured the subcutaneous tissue onto the flexor-pronator mass to keep it completely in a lax position.

### 4:16 Incision for Distal Transfer.

For the distal exposure, we did an approximately 8-cm incision that exposed the distal forearm to just proximal to the wrist crease.

### 4:31 Isolating the Ulnar Neurovascular Bundle and Anterior Interosseous Nerve.

We identified the flexor carpi ulnaris muscle as a landmark and retracted it medially to expose the ulnar neurovascular bundle.

### 4:41 Identifying Anterior Interosseous Nerve.

We then went lateral to this and retracted the flexor muscles and tendons to expose the pronator quadratus muscle and the anterior interosseous nerve along with its vessels.

### 4:58 Identifying the Branches of the Distal Ulnar Nerve and Performing Internal Neurolysis.

We next identified the ulnar nerve dorsal sensory branch, and this allowed it to be a landmark to expose the sensory aspect of the ulnar nerve most medially going to the dorsal cutaneous branch, the main trunk of the sensory nerve most laterally, with the motor branch in the middle. We then performed an internal neurolysis doing an epineurotomy and then separating, first without the microscope and then with the microscope, the sensory dorsal cutaneous branch, which is seen lowermost in this video, the middle part being the motor branch, and then the main trunk of the sensory being most lateral, and that is the one that is closest to the anterior interosseous nerve, as you can see there.^1^

### 5:50 AIN to Ulnar Motor Branch RETS Transfer.

We have gained length on the anterior interosseous nerve distally by taking down some of the pronator quadratus muscle, which we did with Bovie as well as with coagulation and then incision of the muscle. This allowed us to gain great length of the pronator quadratus branch so that when we cut it distally, we could bring it up without tension to the ulnar nerve motor branch. When we stimulate the AIN, we can see that there is robust contraction of the pronator quadratus. When we stimulate the ulnar nerve motor branch, we see there is no contraction of hand intrinsics. Under the operating microscope, we then used a sharp technique to create a perineurial window^1,5^ overlying the ulnar motor branch. We then brought the distal AIN in approximation to this. We used an 8-0 monofilament suture for the end-to-side repair of the distal AIN within the perineurial window to the side of the ulnar motor branch. Usually, two to three sutures are sufficient for this, and these are done so that the repair is under no tension. We then used Tisseel fibrin glue to reinforce the repair and then applied a generous amount of fibrin glue to reinforce the entire incision.

## Disclosures

The authors report no conflict of interest concerning the materials or methods used in this study or the findings specified in this publication.

## Author Contributions

Primary surgeon: Midha. Assistant surgeon: Pathiyil, Elzinga, Umansky. Editing and drafting the video and abstract: Midha, Pathiyil, Umansky. Critically revising the work: Midha, Pathiyil, Elzinga. Reviewed submitted version of the work: Midha, Elzinga, Umansky. Approved the final version of the work on behalf of all authors: Midha. Supervision: Midha, Elzinga.

## Supplemental Information

### Patient Informed Consent

The necessary patient informed consent was obtained in this study.
